# Functional assembly of mammalian cochlear hair cells

**DOI:** 10.1113/expphysiol.2011.059303

**Published:** 2011-12-05

**Authors:** Walter Marcotti

**Affiliations:** Department of Biomedical Science, University of SheffieldSheffield S10 2TN, UK

## Abstract

Hair cells in the mammalian inner ear convert sound into electrical signals that are relayed to the nervous system by the chemical neurotransmitter glutamate. Electrical information encoding sound is then passed through the central nervous system to the higher auditory centres in the brain, where it is used to construct a temporally and spatially accurate representation of the auditory landscape. To achieve this, hair cells must encode fundamental properties of sound stimuli at extremely high rates, not only during mechano-electrical transduction, which occurs in the hair bundles at the cell apex, but also during electrochemical transduction at the specialized ribbon synapses at the cell base. How is the development of such a sophisticated cell regulated? More specifically, to what extent does physiological activity contribute to the progression of the intrinsic genetic programmes that drive cell differentiation? Hair cell differentiation takes about 3 weeks in most rodents, from terminal mitosis during embryonic development to the onset of hearing around 2 weeks after birth. Until recent years, most of the molecules involved in hair cell development and function were unknown, which was mainly due to difficulties in working with the mammalian cochlea and the very small number of hair cells, about 16,000 in humans, present in the auditory organ. Recent advances in the ability to record from the acutely isolated cochlea maintained in near-physiological conditions, combined with the use of genetically modified mouse models, has allowed the identification of several proteins and molecular mechanisms that are crucial for the maturation and function of hair cells. In this article, I highlight recent findings from my laboratory that have furthered our understanding of how developing hair cells acquire the remarkable sensitivity of adult auditory sensory receptors.

Mammalian auditory hair cells are the primary sensory cells that detect sound. They are aligned in rows along a coiled sensory epithelium, the organ of Corti, housed within the snail-like cochlea. The cochlea is part of the inner ear, which also contains the five sensory organs of the vestibular system (Fig. 1A), and it is located at the base of the skull, protected within the hard temporal bone. The human cochlea has a remarkable range of sensitivity, detecting frequencies of 20–20,000 Hz and sound intensities from the soft click of a pin dropping to the roar of a jet engine.

**Figure 1:**
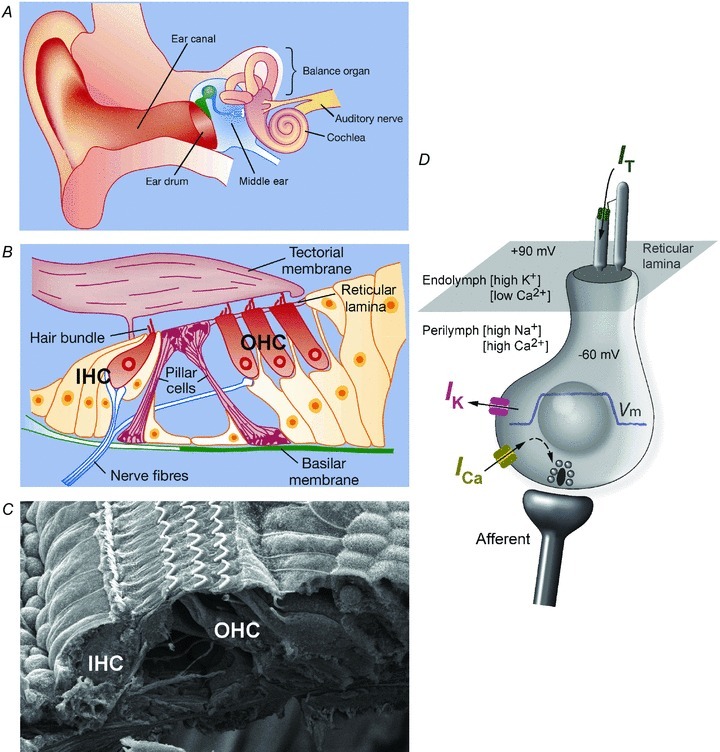
The mammalian ear *A*, diagram of the human ear. *B*, diagram depicting a transverse section of the sensory part of the mammalian cochlea, the organ of Corti, which is located in between two extracellular matrices, namely the basilar membrane and the tectorial membrane. Two types of sensory cells are present, namely inner hair cells (IHCs; one row) and outer hair cells (OHCs; three rows). The IHCs are mainly contacted by auditory afferent nerve fibres and are responsible for sending information to the brain. The OHCs are mainly innervated by inhibitory efferent fibres, which influence their mechanical responses. Drawings in *A* and *B* are modified from Holley MC (2000)*Nature* 405, 130–133. *C*, scanning electron micrograph of the organ of Corti, showing the stereociliary bundle projecting from the apical part of IHCs and OHCs. Image courtesy of D. N. Furness, Keele University, Keele, UK. *D*, schematic diagram depicting the basic physiology of adult IHCs. The reticular lamina separates the mechanosensory hair bundles (hair cell apical pole) from the hair cell basolateral membrane so that solutions with different ion compositions are present, namely endolymph and perilymph. The transducer current (*I*_T_) is driven by a large electrical driving force of about 150 mV, which is determined by the endocochlear potential (about +90 mV) and the hair cell resting membrane potential (about −60 mV). The transducer current causes IHCs to depolarize, which generate a receptor potential (blue trace) and the Ca^2+^-induced fusion of synaptic vesicles at the presynaptic site of the cell, leading to the release of the neurotransmitter glutamate that activates the auditory afferent fibres.

To stimulate hair cells, sound pressure waves enter the outer ear and cause vibrations of the eardrum. They are then transmitted through the middle ear bones to the much smaller ‘oval window’ and thus into the fluid within the cochlear duct (Fig. 1A). Oscillations of fluid pressure cause displacements in the basilar membrane, a ribbon-like collagenous sheet that lies along the cochlea and that supports the organ of Corti (Fig. 1B). The mechanical properties of the basilar membrane mean that it is more sensitive to high-frequency oscillations at the base of the cochlea and to low frequencies at the apex. The geometry of the organ of Corti and the disposition of another collagenous structure, the tectorial membrane (Fig. 1B), ensure that when the basilar membrane vibrates it causes lateral deflection of the mechanosensory hair bundles (Fig. 1B and C) that are the characteristic feature of hair cells (Petit & Richardson, 2009; Schwander *et al*. 2010).

There are two types of hair cells, normally arranged in four rows along the organ of Corti (Fig. 1B and C). The inner hair cells (IHCs) form a single row and act as the primary sensory receptors, the lack of which causes profound hearing loss. Outer hair cells (OHCs) form the other three rows and function to enhance the sensitivity and frequency selectivity along the cochlea. This is achieved at least in part via voltage-dependent somatic electromotility or cell body contractions (Fettiplace & Hackney, 2006), mediated by an unusual motor protein called prestin (Zheng *et al*. 2000). Prestin is densely packed in the basolateral membrane of OHCs and is able to generate extremely rapid changes in cell length, which are likely to be driven by the receptor potentials of the cell over the full frequency range of the mammalian ear (Johnson *et al*. 2011*a*).

This review focuses primarily on IHCs. As in all hair cells, their mechanosensory bundles are composed of stereocilia that resemble large microvilli arranged in ordered rows that project from the cell surface (Fig. 1B–D). The morphological and biophysical properties of IHCs change progressively along the cochlea, ensuring that each cell is tuned to respond best to specific sound frequencies. For example, the hair bundles are longest in the low-frequency cells at the apex of the cochlea and shortest in the high-frequency cells at the base (Fettiplace & Hackney, 2006). Deflection of the hair bundle opens mechanosensitive ion channels that carry a depolarizing inward current, which generates sustained and graded receptor potentials within the hair cells (Fig. 1D). These receptor potentials are responsible for modulating the fusion of synaptic vesicles and release of glutamate from specialized presynaptic structures called ribbon synapses (Glowatzki *et al*. 2002). As hair cells process an extraordinary amount of information with great accuracy and at extremely high rates, the mechanisms of mechano-electrical transduction in the hair bundle and of electrochemical transduction at the ribbon synapse must be very closely integrated. Whilst the question of how are they put together during development is fascinating in its own right, it is also relevant to the challenge of stimulating regeneration of hair cells to recover hearing loss.

Hair cells take about 3 weeks to differentiate through the earliest days of life. In most rodents, before the onset of hearing some 12 days after birth the hair cells follow a tightly regulated developmental programme, during which they acquire and/or eliminate different combinations of ion channels and other membrane proteins. Hair cells must remain viable at all stages of development, and their own intrinsic physiological activity guides the nature and timing of critical steps in the formation of the hair bundles and synaptic machinery. Several molecular mechanisms responsible for co-ordinating the ordered assembly of these structures are now being uncovered through studies on knockout mice and mouse models of hearing loss. This review describes mouse models that reveal the close relationship between the hair bundles and synaptic machinery during IHC differentiation and focuses on the influence of early spontaneous electrical activity.

## Experimental set-up and electrophysiology

**Figure 2:**
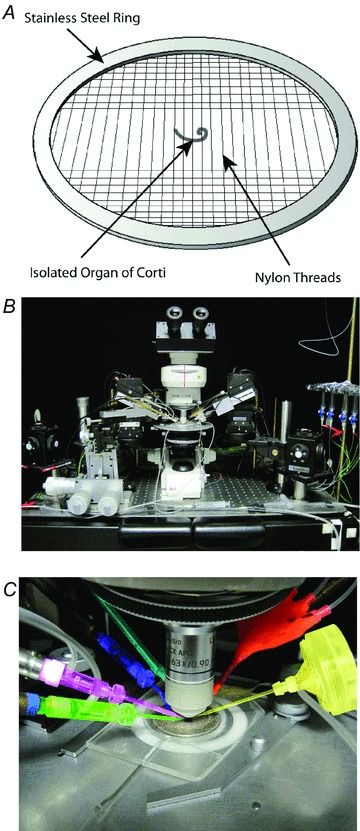
Single-cell electrophysiological set-up *A*, the acutely dissected cochlea is positioned under a nylon mesh fixed to a stainless-steel ring in the recording chamber. *B*, the chamber is placed onto a rotating stage of an upright microscope and is continuously perfused with physiological extracellular solution. *C*, view of the recording chamber and rotating stage, which has been modified so that its centre can be heated to keep the preparation at around body temperature. The different elements used for experiments are as follows: patch pipette to record voltage and current responses from hair cells (pink); cleaning pipette to gain access to hair cells (green); earth electrode (blue); thermocouple to maintain a constant temperature throughout the recordings (turquoise); topical perfusion system used to deliver drugs (red); and a piezo-driven fluid jet used to deflect the stereociliary bundles with a stream of solution, thus mimicking *in vivo* stimulation (yellow). The original design of the temperature system and fluid jet were from C. J. Kros (University of Sussex, Brighton, UK).

Single-cell patch-clamp recordings were performed from hair cells positioned along the acutely dissected organs of Corti of altricial rodents at different stages of development. The dissected organ of Corti was transferred to a recording chamber containing extracellular solution and immobilized with a nylon mesh fixed to a stainless-steel ring (Fig. 2). The chamber was then mounted on a rotating stage of an upright microscope. The rotating stage allowed recordings to be made from hair cells at different characteristic frequencies along the same cochlea. The centre of the microscope stage was modified so that small resistors, which warm up when current is passed through them, could be fixed to the underside in order to perform recordings at near body temperature (35–37°C). Constant temperature was maintained throughout the duration of the experiment via a feedback loop provided by a thermocouple. A piezo-driven fluid jet was used for displacing the stereociliary hair bundles to activate the mechano-electrical transducer channels.

## Hair cell stereociliary bundle development

**Figure 3:**
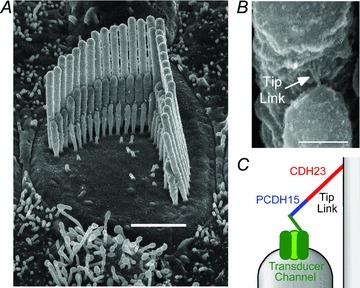
Mature hair bundle morphology of a cochlear hair cell *A*, scanning electron micrograph showing the hair bundle from an adult guinea-pig apical coil outer hair cell. Note the characteristic staircase structure composed by rows of stereocilia of decreasing height. Scale bar represents 2 μm. *B*, high-magnification scanning electron micrograph view of a tip link connecting adjacent stereocilia from an adult rat IHC. Scale bar represents 200 nm. Images in *A* and *B* are courtesy of D. N. Furness, Keele University, Keele, UK. *C*, schematic drawing of the tip link structure connecting the mechano-electrical transducer channel to the adjacent taller stereocilia. Abbreviations: CDH23, cadherin-23; and PCDH15, protocadherin-15.

The development of the hair bundle is a highly regulated process that determines not only the lengths of individual stereocilia on each cell but also the gradation of length along the cochlea. Stereocilia in mammals are typically organized in three or four rows of decreasing height to form a staircase structure (Fig. 3A). Individual stereocilia are coupled within and between rows by extracellular links of several types (Petit & Richardson, 2009). The transduction of acoustic stimuli into electrical signals relies on the orientation of the stereociliary hair bundles along the axis of mechanosensitivity (Fig. 1C), which is critical for the optimal opening of transducer channels (Fettiplace & Hackney, 2006). The gating of transducer channels is thought to depend on the stretching of fibrous links, called tip links (Fig. 3B),which are formed by two Ca^2+^-dependent transmembrane proteins positioned in series (Fig. 3C), cadherin-23 and protocadherin-15 (Schwander *et al.* 2010). Although the nature of the mechano-electrical transducer channel in hair cells is still elusive, there is now evidence indicating its possible localization at the tip of the shorter and middle stereocilia (Beurg *et al*. 2009). In the absence of sound stimulation, the transducer channels are partly open, owing to resting tension within the hair bundle. Sound stimulation displaces hair bundles towards the taller stereocilia, stretching the tip links and causing the opening of transducer channels, whereas movement in the opposite direction closes the channels.

**Figure 4:**
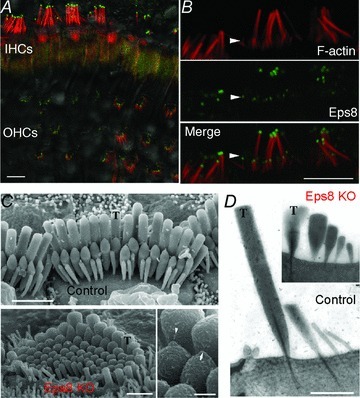
The actin-binding protein, Eps8, regulates hair bundle morphology *A*, IHC and OHC stereocilia showing immunostaining for F-actin (red) and Eps8 (green) from adult mice using confocal microscopy. Fluorescence image is superimposed on the differential interference contrast image. *B*, high-magnification image of IHC stereocilia showing that Eps8 is localized in the tips of stereocilia. Scale bars in *A* and *B* represent 5 μm. *C*, scanning electron micrograph showing the hair bundle structure in adult IHCs from control (top panel) and Eps8 knockout mice (Eps8 KO; bottom left panel). Taller stereocilia are indicated by T. Control hair bundles are normally composed of three rows of stereocilia. In Eps8 knockout mice, hair bundles are disorganized and shorter and additional rows of stereocilia are present compared with wild-type mice. Bottom right panel shows the presence of tip links between stereocilia (arrow and arrowhead; Eps8 knockout IHC), which are required for gating the mechanoelectrical transducer channels. Scale bars represent 2 μm (top panel), 1 μm (bottom left panel) and 250 nm (bottom right panel). *D*, transmission electron micrographs showing the stereociliary bundle from control and Eps8 knockout adult IHCs. Note that in this case the Eps8 KO IHC shows an extra row of stereocilia compared with the control cell. Scale bar represents 1 μm. Figure modified from Zampini *et al.* (2011).

The height of stereocilia within each row is similar not only within a single hair bundle but also between bundles on adjacent hair cells. The length of stereocilia is tightly controlled by regulation of the polymerization and depolymerization of the actin filament core (Tinley *et al*. 1992). At birth, the stereociliary bundles are immature in terms of their morphological appearance and their ability to transduce mechanical stimuli into electrical signals. As with most of the other morphological and biophysical characteristics of adult hair cells, hair bundle formation and the associated mechanical sensitivity develop primarily during the first two postnatal weeks (Schwander *et al*. 2010). Several stereociliary proteins, including whirlin, espin and the unconventional myosins VIIa and XVa, are crucial for the normal development and/or maintenance of hair bundle structure and function, and their mutation causes deafness in both mice and humans (Petit & Richardson, 2009; Schwander *et al*. 2010). However, none of these proteins is known to regulate actin polymerization directly, which is required for elongation of stereocilia. Recent studies have identified three new stereociliary proteins that are able to control actin elongation and maintenance directly via their actin barbed end capping and/or bundling activity, namely twinfilin 2 (Peng *et al.* 2009), gelsolin (Mburu *et al.* 2010) and the epidermal growth factor receptor pathway substrate 8 (Eps8; Manor *et al.* 2011; Zampini *et al.* 2011). Twinfilin 2 and gelsolin are specifically expressed at the tips of the short and mid-length stereocilia and have been shown to inhibit the growth of actin filaments (and consequently stereocilia) via their actin capping activity (Peng *et al.* 2009; Mburu *et al.* 2010). In contrast, Eps8 is present at the tips of all stereocilia and appears to favour elongation of stereocilia, because in hair cells from Eps8 knockout mice the hair bundles were abnormally short (Fig. 4). Despite the shorter stereocilia, tip links were present (Fig. 4C), and a large mechanotransducer current was recorded in response to bundle displacement with large force stimuli (Zampini *et al.* 2011). These findings indicate that Eps8 is required for the normal growth and maintenance of the stereocilia but not for the intrinsic biophysical properties of the transducer channel. However, the short hair bundles of Eps8 knockout mice are unlikely to be displaced effectively *in vivo* by physiological sound stimuli, precluding the normal function of the transducer channels. An additional interesting physiological consequence of the absence of Eps8 was the failure in the maturation of the synaptic vesicle fusion process at the base of IHCs (Zampini *et al.* 2011), which highlights possible functional integrations between the development of the mechano-electrical transduction in the hair bundle and electrochemical transduction at the ribbon synapse. The failure in cochlear hair cell development is the reason why Eps8 knockout mice are profoundly deaf (Zampini *et al.* 2011).

## Spontaneous electrical activity in prehearing IHCs

**Figure 5:**
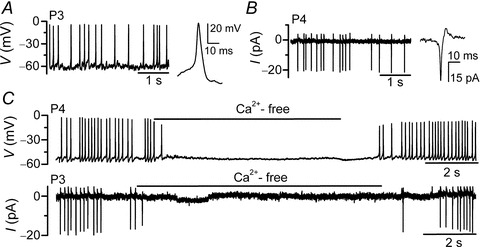
Spontaneous Ca^2+^-dependent action potentials in IHCs *A* and *B*, spontaneous electrical activity in immature IHCs recorded with whole-cell current clamp (*A*) and cell-attached voltage clamp (*B*). *C*, recordings of whole-cell (top panels) and cell-attached spontaneous action potentials (bottom panels) in IHCs with the superfusion of a Ca^2+^-free solution. P3 and P4 indicate postnatal day 3 and 4, respectively. Figure modified from Johnson *et al.* (2011*b*).

**Figure 6:**
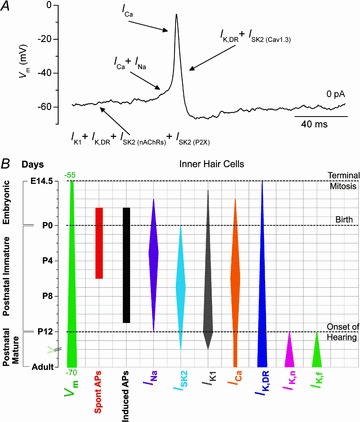
Role of various membrane currents in shaping IHC action potentials *A*, an action potential recorded from a spontaneously active postnatal day 3 IHC. The different phases of an action potential are primarily determined by different basolateral currents expressed in immature IHCs (indicated by arrows). The IHC resting membrane potential is mainly set by the interplay of the following three currents: a classical delayed rectifier K^+^ current (*I*_K,DR_; Marcotti *et al.* 2003*a*); an inward rectifier K^+^ current (*I*_K1_; Marcotti *et al.* 1999); and a small-conductance Ca^2+^-activated K^+^ current (*I*_SK2_) gated by Ca^2+^ influx through α9α10 nicotinic acetylcholine receptors (*I*_SK2(nAChRs)_; Marcotti *et al.* 2003*a*; Johnson *et al.* 2007) or purinergic receptors (*I*_SK2(PX2)_; Johnson *et al.* 2011*b*). While the Ca^2+^ current carried by Ca_V_1.3 Ca^2+^ channels is essential for the generation of action potentials (*I*_Ca_; Marcotti *et al.* 2003*b*; Johnson *et al.* 2011*b*), the Na^+^ current is required for speeding up the time to spike threshold (*I*_Na_; Marcotti *et al.* 2003*b*). Finally, IHC repolarization after an upstroke is determined by both *I*_K,DR_ and the SK2 current, which (different from that described above) is activated by Ca^2+^ flowing through closely colocalized Ca_V_1.3 Ca^2+^ channels (*I*_SK2(Cav1.3)_; Marcotti *et al.* 2004*b*; Johnson *et al.* 2007). *B*, developmental changes in the expression of some IHC biophysical properties (mainly from mice). The width of the vertical bars provides an indication of the developmental change in the size of the currents or membrane potential (*V*_m_) in apical IHCs. The day of birth (P0) corresponds to embryonic day 19.5 (E19.5).

While hair bundle architecture and the biophysical properties of the mechano-electrical transducer channel gradually develop, the immature IHCs are not electrically silent, passively waiting for a sound stimulus. Instead, IHCs generate spontaneous action potentials. These action potentials are thought to guide the development of the immature mammalian cochlea before the onset of sensory-driven activity. Although action potentials in IHCs are mainly carried by Ca^2+^ (Fig. 5; see also Marcotti *et al.* 2003*b*; Johnson & Marcotti, 2008; Johnson *et al.* 2011*b*), as opposed to the Na^+^ spikes found in most neurons, their shape is modulated by several transiently expressed inward and outward currents (Fig. 6*A*). The expression of these currents varies as a function of IHC development (Fig. 6B) and position along the cochlea, causing a corresponding change in the action potential waveform and frequency (Marcotti *et al.* 2003*a*,*b*; Johnson *et al.* 2011*b*). Transient increases of intracellular Ca^2+^, such as those associated with spontaneous action potentials, are known to influence gene expression (Moody & Bosma, 2005), synaptic maturation and refinement of neural circuits (Zhang & Poo, 2001). If spontaneous action potentials were proved to influence cochlear development directly, then changes in their frequency and/or pattern could drive different developmental signals within the cochlea. An instructive role for this activity in the cochlea, similar to that found in the immature visual system (e.g. McLaughlin *et al*. 2003), could occur during a ‘critical period’ of IHC development. Such a critical period is likely to exist because, for example, the surgical removal of the cochlea within the first postnatal week, as opposed to later stages, resulted in a considerable loss of cochlear nucleus neurons in adult mice (Mostafapour *et al*. 2000).

Action potential activity can be elicited in mammalian cochlear IHCs from just before the day of birth up to the onset of hearing (Kros *et al.* 1998; Marcotti *et al.* 2003*a*). However, IHCs fire spontaneous action potentials mainly during the first postnatal week, because during this period their resting membrane potential resides near the action potential threshold [membrane potential (*V*_m_) −55 to −60 mV; Fig. 6B; Marcotti *et al.* 2003*a*; Johnson *et al.* 2011*b*]. This time window corresponds to the critical period highlighted from *in vivo* experiments (Mostafapour *et al*. 2000). During the second postnatal week, the *V*_m_ of IHCs seems to become more hyperpolarized (Fig. 6B), which is mainly due to a larger contribution of the inward rectifier K^+^ current, *I*_K1_ (Marcotti *et al.* 1999). The more hyperpolarized *V*_m_ of these older IHCs indicates that repetitive action potential activity can only be elicited by depolarizing external stimuli, such as those associated with the activation of purinergic receptors (Tritsch *et al.* 2007). From about the onset of hearing (postnatal day 12; Fig. 6B), the concomitant expression of the rapidly activating large-conductance Ca^2+^-activated K^+^ current (*I*_Kf_; Kros *et al.* 1998; Marcotti *et al.* 2004*a*) and the negatively activating delayed rectifier, *I*_K,n_, carried by KCNQ4 channels (Marcotti *et al.* 2003*a*), completely prevents action potential activity in mature IHCs.

**Figure 7:**
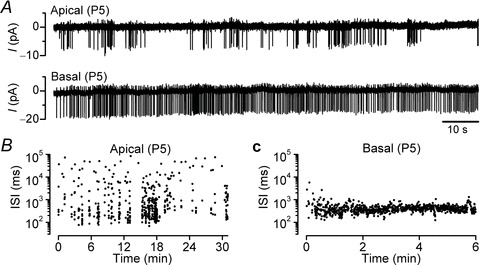
Spontaneous action potential activity in IHCs is patterned along the cochlea *A*, spontaneous spiking activity from an apical (top) and a basal IHC (bottom) using cell-attached recordings. *B* and *C*, interspike intervals (ISIs) as a function of recording time from the IHCs shown in *A*. Note that in apical IHCs, the presence of periods of silent/reduced electrical activity indicates a variable (i.e. bursting-like) spiking rate in these cells, which is different from the more continuously firing basal cells. Figure modified from Johnson *et al.* (2011*b*).

During the first postnatal week, the frequency and pattern of spontaneous firing activity differ progressively along the mouse and rat cochlea (Johnson *et al.* 2011*b*), with apical IHCs showing a bursting-like pattern and basal cells a more regular pattern with a higher mean frequency (Fig. 7). This position-dependent pattern of IHC action potential activity is likely to be determined by the more hyperpolarized *V*_m_ in apical IHCs compared with that of basal cells (Johnson *et al.* 2011*b*). An important question is: what is the underlying mechanism responsible for the different pattern of action potential activity along the cochlea? The fine-tuning of the IHC *V*_m_ is determined, in addition to the various membrane currents (see Fig. 6), by the opening of α9α10 nicotinic acetylcholine receptors (α9α10n AChRs), which are activated by the efferent neurotransmitter, ACh (Elgoyhen *et al.* 2001; Maison *et al.* 2002), and purinergic receptors activated by ATP released by supporting cells (Housley *et al.* 2006; Tritsch *et al.* 2007). The influx of Ca^2+^ into immature IHCs through α9α10n AChRs or purinergic receptors causes the activation of the hyperpolarizing potassium current, *I*_SK2_, thus exerting an inhibitory effect on the voltage responses of the cells (Glowatzki *et al.* 2000; Marcotti *et al.* 2004*b*; Johnson *et al.* 2011*b*). The different patterns of IHC action potentials could be attributed to differences in the spontaneous release of ACh from efferent fibres and ATP from supporting cells. The balance between the action of ACh and ATP keeps the *V*_m_ of apical IHCs slightly, but significantly, more hyperpolarized than in basal cells (Johnson *et al.* 2011*b*). The presence of a position-dependent firing pattern would be appropriate to instruct the maturation of IHCs (Housley *et al.* 2006) as well as the refinement of auditory connections and sensory maps (Kandler *et al.* 2009) before the onset of hearing. In particular, it could influence the biophysical and morphological differentiation of the synaptic machinery of the IHC, which is known to change as a function of development and cell position along the cochlea (Sobkowicz *et al.* 1982; Johnson *et al.* 2008, 2009).

## Functional maturation of the synaptic machinery in IHCs

Mature sensory synapses, such as those in the auditory, vestibular and visual systems, are designed to encode graded receptor potentials over a wide range of sensory information and be able to sustain neurotransmitter release for prolonged periods of time. This differs from conventional synapses and immature auditory synapses, because both encode all-or-nothing action potential activity. In the adult mammalian cochlea, synaptic transmission is made even more challenging by the fact that sound frequencies and intensities have to be transmitted to the auditory afferent fibres with exquisite temporal precision essential for sound perception and localization (Fuchs, 2005). The activity of each afferent fibre is driven by the release of glutamate from a single IHC presynaptic specialization called the synaptic ribbon (Glowatzki *et al.* 2002). Ribbon synapses, which are also found in other sensory cells, such as photoreceptors and vestibular hair cells, are specialized electron-dense organelles that tether a large pool of releasable synaptic vesicles and are thought to be important for co-ordinated vesicle release (Matthews & Fuchs, 2010). The fusion of vesicles to the IHC presynaptic membrane is triggered by Ca^2+^ flowing through one or very few Ca_V_1.3 Ca^2+^ channels positioned within a few tens of nanometres from each docked vesicle (Brandt *et al.* 2003; Johnson *et al.* 2008; Zampini *et al.* 2010).

**Figure 8:**
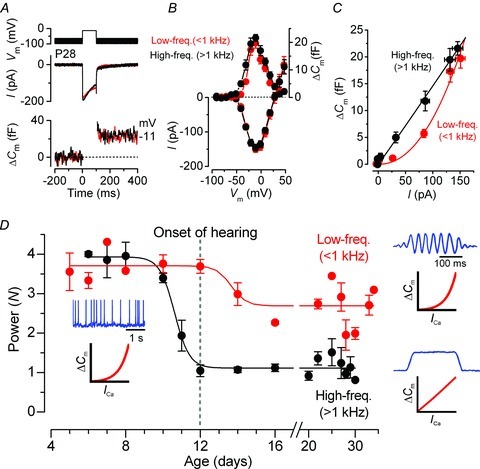
Calcium dependence of neurotransmitter release in developing IHCs *A*, Ca^2+^ current (*I*_Ca_) and changes in membrane capacitance (Δ*C*_m_) from low-frequency (<1 kHz, apical coil; red) and high-frequency gerbil cochlear IHCs (>1 kHz, basal coil; black). Recordings were obtained in response to 100 ms voltage steps from −81 mV, in 10 mV nominal increments. For clarity, only responses near the peak *I*_Ca_ (−11 mV) are shown. *B*, average peak *I*–*V* and Δ*C*_m_–*V* curves in apical and basal IHCs. *C*, synaptic transfer functions obtained by plotting average Δ*C*_m_ against the corresponding *I*_Ca_ between −71 and −11 mV from *B*. Data shown in *C* were approximated using a power function, Δ*C*_m_=*cI^N^*_Ca_ (eqn 1), where *c* is a scaling coefficient and the exponent *N* is the power. *A–C* are modified from Johnson *et al.* (2008). *D*, developmental changes in *N* from both apical (low-frequency, ∼300 Hz) and basal gerbil IHCs (high-frequency, ∼30 kHz) as a function of postnatal day (modified from Johnson *et al.* 2009). Insets show the characteristic receptor potentials (blue) and exocytotic Ca^2+^ dependence curves (red) of IHCs at different stages of development and position along the cochlea. Receptor potentials of adult IHCs are drawings that approximate results from Palmer & Russell (1986).

At around the onset of hearing, IHCs stop firing slow and repetitive action potentials and instead respond with rapid, graded and sustained receptor potentials. Adult receptor potentials are required to follow high-frequency sound continuously as well as the characteristics of the auditory stimulus, including intensity and duration. Therefore, the biophysical properties of the synaptic machinery of the IHC have to be appropriate to encode accurately the different receptor potentials characteristic of immature and posthearing cells. Recent studies have indicated that auditory ribbon synapses may be able to follow the different receptor potentials accurately by changing the relationship between Ca^2+^ influx and neurotransmitter release (i.e. Ca^2+^ dependence) during development (Johnson *et al.* 2008, 2009). Neurotransmitter release from immature IHCs shows, similar to conventional synapses (Augustine & Charlton, 1986), a high-order Ca^2+^ dependence (power of four; i.e. dependent on the fourth power of the local Ca^2+^ concentration; Fig. 8), which ensures that exocytosis mainly occurs during spikes rather than during interspike intervals. Adult IHCs respond differently to membrane depolarization depending on their position along the cochlea. Low-frequency IHCs (mainly those responding to sound frequencies below 1 kHz) are phase locked to sound stimulation such that their receptor potentials exhibit a pronounced phasic component representing the sound frequency. In contrast, the IHC membrane filtering prevents phase locking in high-frequency IHCs (mainly responding to sound above 1 kHz) so that receptor potentials are graded and sustained to better represent sound intensity and stimulus duration (Palmer & Russell, 1986). The different receptor potential characteristics of adult IHCs are reflected in the properties of exocytosis, with low-frequency cells having a high-order Ca^2+^ dependence (power of two to three; Fig. 8) and high-frequency cells showing a linear dependence on Ca^2+^ influx (power of approximately one; Fig. 8). The steeper Ca^2+^ dependence in low-frequency cells could accentuate the phasic (time-locked) receptor potentials, which resemble to some extent the action potentials of immature IHCs. Hair cells from lower vertebrates, which mainly operate at low frequencies (<1 kHz) and exhibit a large phasic component in their receptor potential, seem to have a linear exocytosis Ca^2+^ dependence (Schnee *et* al. 2005; Keen & Hudspeth, 2006); however, a higher order of exocytotic Ca^2+^ dependence was seen for voltage steps that elicited small Ca^2+^ currents, which could reflect the intrinsic characteristic of their synaptic machinery (Keen & Hudspeth, 2006). In mammals, a linear exocytotic Ca^2+^ dependence was only observed in high-frequency IHCs (>1 kHz), which is likely to allow them to respond efficiently to both small and large sustained changes in membrane potential, and thus enable them to discriminate sound intensity over a wide dynamic range (Johnson *et al.* 2008, 2009). The observed dependence of exocytosis on Ca^2+^ appears to be an intrinsic property of the IHC synapse (Johnson *et al.* 2008, 2009), suggesting a possible differential expression of synaptic proteins, such as Ca^2+^-sensing molecules, as a function of position along the adult cochlea and development. The discovery of the Ca^2+^ sensor(s) triggering exocytosis, as well as other synaptic proteins involved in neurotransmitter release at IHC ribbon synapses, has been one of the major challenges in hearing research for at least a decade.

## Synaptic proteins determining the exocytotic Ca^2+^ dependence at auditory ribbon synapses

**Figure 9:**
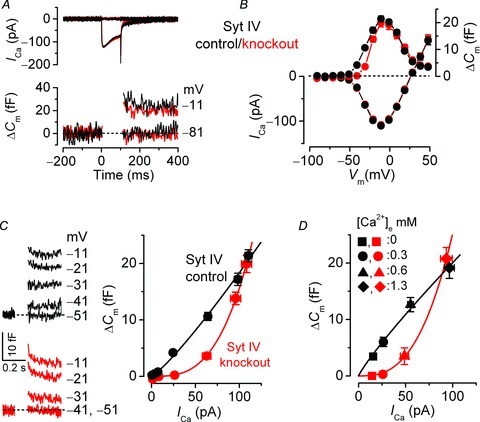
Calcium dependence of neurotransmitter release in adult IHCs from control and Syt IV knockout mice *A* and *B*, Ca^2+^ current (*I*_Ca_) and changes in membrane capacitance (Δ*C*_m_) from adult IHCs in response to voltage steps, in 10 mV increments, from the holding potential of −81 mV. Extracellular Ca^2+^ concentration, 1.3 mm. In *A*, only peak responses and those at −81 mV are shown. *C*, left panels show average Δ*C*_m_ traces from control and knockout IHCs; right panel shows the synaptic transfer curves obtained by plotting Δ*C*_m_ against *I*_Ca_ for voltage steps between −71 and −11 mV (*B*). Fits are according to eqn (1) (see Fig. 8). *D*, synaptic transfer curves, similar to those shown in *C*, but obtained by plotting the maximal *I*_Ca_ and Δ*C*_m_ values during the application of different extracellular Ca^2+^ concentrations. Figure modified from Johnson *et al.* (2010).

The molecular composition of the exocytotic machinery of auditory hair cells differs not only from that present at conventional synapses (Pang & Südhof, 2010) but also from that of other ribbon synapses, such as those in photoreceptors (Zanazzi & Matthews, 2009), especially in the types of synaptic proteins triggering vesicle fusion. For example, hair cell ribbon synapses lack classical molecules involved in exocytosis, such as synaptophysins and synapsins (Safieddine & Wenthold, 1999) and the neuronal SNARE proteins (Nouvian *et al.* 2011), which catalyse the fusion of the synaptic vesicle and plasma membrane (Pang & Südhof, 2010). In contrast, IHC synapses express the ribbon specific protein, RIBEYE (Khimich *et al.* 2005), and the Ca^2+^-binding protein, otoferlin, which has been proposed as the main Ca^2+^ sensor triggering neurotransmitter release in these cells (Roux *et al.* 2006); however, recent evidence has indicated that otoferlin is involved in synaptic vesicle replenishment (Johnson *et al.* 2010; Pangrsic *et al.* 2010) but its role in vesicle fusion is less clear. Another group of Ca^2+^-binding proteins known to trigger exocytosis are the synaptotagmins. Synaptotagmins are a large family of synaptic vesicle proteins that, through their C2 domains, are able to interact with other known synaptic proteins and, in the majority of cases, bind Ca^2+^ directly (Südhof, 2002), thus determining the Ca^2+^ co-operativity of exocytosis (via Ca^2+^ binding to the C2A domain; Shin *et al.* 2009). Synaptotagmins (Syt) I and II are the classical Ca^2+^-sensing triggers for vesicle fusion found at conventional synapses, although their presence in hair cells remains debatable. Some studies promote their role (Syt I, Johnson *et al.* 2010; Beurg *et al.* 2011; and Syt II, Johnson *et al.* 2010), while others exclude their involvement in auditory ribbon synapses (Syt I, Reisinger *et al.* 2011; and Syt II, Beurg *et al.* 2011; Reisinger *et al.* 2011). The identity of the molecular composition of ribbon synapses remains a major challenge. However, one unconventional synaptotagmin, Syt IV, which is unable to bind Ca^2+^ at its C2A domain (Südhof, 2002), has recently been shown to be expressed in high-frequency adult gerbil IHCs but not in low-frequency adult or immature cells (Johnson *et al.* 2010). Thus, the presence of Syt IV appears to be directly correlated with the linear exocytotic Ca^2+^ dependence observed in high-frequency auditory hair cells. These morphological observations were confirmed by electrophysiological recordings from Syt IV knockout mice (Johnson *et al.* 2010), showing that in the absence of Syt IV the normal developmental linearization of the exocytotic Ca^2+^ dependence (Fig. 8B) fails to occur in adult high-frequency IHCs (Fig. 9). A conserved property among Syt isoforms is their ability to form hetero-oligomers (Chapman *et al.* 1998), resulting in a wide range of synaptic Ca^2+^ sensitivities (Südhof, 2002). Therefore, the linearization in the exocytotic Ca^2+^ dependence in mature IHCs could arise from the interaction of Syt IV with additional Ca^2+^-sensing proteins, such as other Syt isoforms or otoferlin. This provides a unique role for Syt IV in tuning/regulating cell physiological responses (Johnson *et al.* 2010).

## Control of mammalian cochlear development: intrinsic genetic programme and/or early sensory-independent electrical activity?

**Figure 10:**
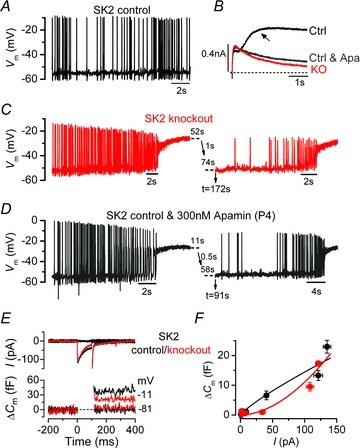
Sustained spiking activity is required for the maturation of the synaptic machinery in adult IHCs *A*, spontaneous action potential activity in current clamp conditions from a control immature IHC. *B*, membrane currents in voltage clamp from control (Ctrl), knockout (KO) and control in the continuous presence of the SK2 channel blocker, apamin (Ctrl & Apa). Recordings were in response to a voltage step from –84 to –34 mV. The SK2 current is only present in the control IHC and is indicated by the arrow. *C*, spontaneous action potentials from an SK2 knockout IHC. In this and the following panel, the time at the break point along the trace (dashed lines) indicates the time omitted from the recording, i.e. 52 s is the time that the cell remained depolarized, 1 s transition to the resting potential and 74 s before trace resumes. *D*, spontaneous action potentials from a control IHC in the continuous presence of apamin. All recordings were obtained at body temperature. *E*, Ca^2+^ current (*I*_Ca_) and changes in membrane capacitance (Δ*C*_m_) from adult IHCs obtained as described in Fig. 9. *F*, synaptic transfer curves obtained by plotting Δ*C*_m_ against *I*_Ca_ for voltage steps between −71 and −11 mV. Fits are according to eqn (1) (see Fig. 8). Figure modified from Johnson *et al.* (2007).

The maturation of the IHC synaptic machinery is a very intricate process that requires the expression of different sets of molecules at different stages of development. It is crucial to understand whether this process is exclusively controlled by a genetic programme or is under the additional influence of the spontaneous action potential activity present in immature IHCs. One way to test the possible involvement of action potentials in the normal maturation of IHCs would be to use a mutant mouse showing an intrinsically altered firing activity, which could be achieved by knocking down an ion channel crucial for shaping their activity. One such current is the small-conductance Ca^2+^-activated K^+^ current, SK2 (Fig. 6), which has been shown to be required for sustaining continuous repetitive spontaneous firing by making the repolarizing phase of each spike more robust. Accordingly, IHCs from SK2 knockout mice exhibit an altered action potential activity (Johnson *et al.* 2007), with periods of repetitive broad spikes in between sustained depolarization (Fig. 10). The functional consequence associated with the altered action potential activity in SK2 knockout mice is a failure of the normal developmental linearization of the exocytotic Ca^2+^ dependence (Fig. 10; see also Johnson *et al.* 2007). The development of all other biophysical properties was indistinguishable between IHCs from control and knockout mice. It is currently unknown whether SK2 knockout mice have normal hearing. These findings, together with the fact that SK2 channels are only expressed in IHCs during immature development (i.e. can only have a functional influence on cochlear development during prehearing stages) and are not directly involved in neurotransmitter release, indicate that the normal linearization of the exocytotic Ca^2+^ dependence in adult IHCs is likely to be controlled by an intracellular developmental signal initiated by a specific frequency and/or pattern of the early spiking activity.

**Figure 11:**
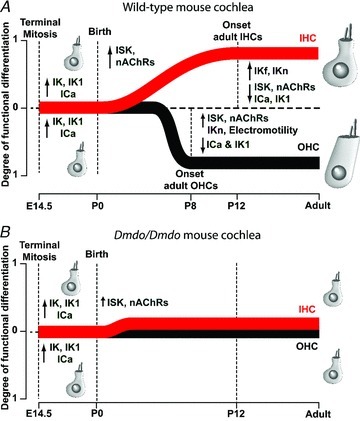
Diminuendo mutant hair cells fail to develop beyond around the day of birth *A*, schematic diagram illustrating the degree of functional differentiation in mammalian cochlear hair cells (1 indicates fully differentiated hair cells; 0 represents electrophysiologically indistinguishable cells). ↑ and ↓ indicate the normal onset and offset of expression, respectively, of ion currents and membrane proteins in control IHCs (red) and OHCs (black). Note that during development, hair cell size increases. The onset of functional maturation is postnatal day (P)12 for IHCs and P8 for OHCs. Before birth, hair cells show qualitatively similar biophysical characteristics. *B*, in diminuendo mutant (*Dmdo*/*Dmdo*) cochleae the normal physiological and morphological development of hair cells stops prematurely (at around the day of birth). Figure modified from Kuhn *et al.* (2011). Definitions of and references for *I*_K1_, *I*_K_, *I*_Ca_, *I*_SK_, *I*_K,n_ and *I*_*K*,f_ in IHCs are given in the main text. For OHCs, for *I*_K_, *I*_K,n_ and electromotility see Marcotti & Kros (1999); for *I*_Ca_ see Michna *et al.* (2003); for *I*_K1_ see Marcotti *et al.* (1999); and for *I*_SK_ see Marcotti *et al.* (2004*b*).

Action potentials alone are unlikely to be sufficient to drive the development of the synaptic machinery, because recent studies, using mice with knocked out the acting binding protein Eps8 (Zampini *et al.* 2011), and mutant mice for the transmembrane protein, Tmc1 (Marcotti *et al.* 2006), showed that the linearization of the exocytotic Ca^2+^ dependence did not occur, despite the fact that immature IHCs exhibited spontaneous spiking activity (Marcotti *et al.* 2006; Zampini *et al.* 2011); therefore, Eps8 and Tmc1 are required for the normal physiological differentiation of hair cells into fully functional sensory receptors. In their absence, hair cell development is arrested prematurely at around the onset of hearing (Marcotti *et al.* 2006; Zampini *et al.* 2011). A different level of control over hair cell development is exerted by the microRNA, miR-96 (Kuhn *et al.* 2011), which is a sensory organ-specific microRNA expressed in the immature mouse cochlea (Lewis *et al.* 2009). In mammals, microRNAs regulate posttranscriptional gene expression programmes by decreasing the level of target mRNA (Guo *et al.* 2010) and are involved in tissue development (Stefani & Slack, 2008). In the diminuendo mouse model for human deafness (Lewis *et al.* 2009), mutation in miR-96 causes a co-ordinated arrest in the normal biophysical and morphological development of cochlear hair cells at around the day of birth (Fig. 11), which is well before that observed in absence of Eps8 or Tmc1. This early break in hair cell development prevents them from functionally differentiating into the two types of receptors in the adult cochlea, namely IHCs and OHCs. Therefore, miR-96 is able to orchestrate one of the most distinctive functional refinements of the mammalian auditory system (Kuhn *et al.* 2011). It appears that genetic control over IHC maturation guides the cells through different crucial ‘check points’ during postnatal development. Once these check points have been passed, IHCs are then able to respond appropriately to the intrinsic electrical activity.

## Conclusion

Over the last decade or so, remarkable progress has been made in our understanding of the molecular mechanisms that regulate auditory development and function, especially at the level of the sensory hair cell. A large part of this progress has been driven by the study of genes linked to deafness (Petit & Richardson, 2009; Schwander *et al.* 2010). The present findings show that the functional assembly of mammalian cochlear hair cells is a very highly co-ordinated process, and understanding it will pose a challenging but exciting task for the future. It requires the combination of an intricate genetic programme that has to be in place before a critical period, during which time the intrinsic electrical activity of the IHC is able to refine development towards the onset of sensory-driven activity. Despite this progress, we are still far from a comprehensive understanding of all the elements required for hair cell development. For example, the nature of the mechano-electrical transducer channel remains elusive, as well as the intracellular cascade controlling hair cell development caused by action potential activity. Moreover, most of the elements and molecular mechanisms that control neurotransmitter release at hair cell ribbon synapses are still largely unknown. It is important to recognize that these apparently separate processes are closely integrated not only in terms of mature function but also through development. Undoubtedly, over the next few years we will be able to further our understanding of how hair cells are built and maintained during immature and adult stages. It is likely that this will be driven by continued investigation into mouse models of hearing loss, which have proved to be very powerful tools for investigating molecular mechanisms of cell function and development.
